# Strengthening social protection for TB patients: Lessons from COVID-19

**DOI:** 10.1371/journal.pgph.0000950

**Published:** 2022-08-24

**Authors:** Poonam Khetrapal Singh

**Affiliations:** Regional Director, WHO South-East Asia Region, New Delhi, India; Stellenbosch University, SOUTH AFRICA

As the world looks to re-embark on its journey towards ending tuberculosis (TB) in the COVID-19 era, the focus is now on the WHO South-East (SE) Asia Region. The Region already has the highest burden of TB among all WHO regions, accounting for nearly half of the global TB deaths even in the pre-pandemic era [1]. It is estimated that in 2020, nearly 4.3 million people fell ill with TB and nearly 700 000 died (excludes HIV+TB mortality). Of the 11 Member States in the WHO SE Asia Region (Bangladesh, Bhutan, Democratic People’s Republic of Korea, India, Indonesia, Maldives, Myanmar, Nepal, Sri Lanka, Thailand and Timor-Leste), six of them are high-TB burden countries. Patient cost surveys in countries of the Region revealed that 30–80% households bear catastrophic costs due to the disease forcing families to sell their assets and pull children out of school [2–4].

Globally, the COVID-19 pandemic impacted TB in several ways–from massive disruption of services to aggravation of determinants of TB [5]. The Region was not spared either: the top two countries contributing to a maximum drop in TB notifications in 2020 are from the South-East Asia Region and another two among the top 10 countries showing the maximum decline. TB deaths showed an upward trend for the first time in more than a decade and are now very close to the levels seen in 2015 [6].

Traditionally, TB care has primarily focused on a biomedical approach strengthening the diagnostic and treatment machinery and capacity building of the health-care professional engaged in the provision of these services. However, accelerating progress towards ending TB requires more than a biomedical approach that is inclusive of addressing underlying comorbidities and social determinants of TB [7], particularly in the wake of the COVID-19 pandemic.

The “Swiss Cheese Model” for Ending TB also illustrates that it is only through multisectoral collaborations that address the personal, societal and health system layers of care that we will be able to end TB. Within each layer of the interventions, there are gaps that lead to negative TB outcomes. COVID-19 has impacted the different layers of TB care presented in the model, thus further increasing the degree and import of adversity. It is only by addressing these multiple layers that we can protect individuals from either TB infection or TB-related mortality [8].

The issue of “missing” TB cases has been discussed even in the pre-COVID era and becomes rather more relevant due to the COVID-19 pandemic-related fall in case notification. With an estimated 20–40% decline in TB case detection in the South-East Asia Region during 2020 due to COVID-19 outbreak, the missing TB cases increased by 70% between 2019 and 2020, from around 1 million to around 1.7 million [1]. The socioeconomic challenges faced by patients contribute to these missing millions [9]. However, the funding outlay for support to TB patients is not enough. As reported to WHO, the Region collectively budgeted about US$ 150 million in 2021 for patient support activities [10]. The Regional Strategic Plan estimates the funding needs for patient support to be nearly US$ 400 million annually [11].

The economic and social disruption caused by the pandemic is devastating, tens of millions of people have fallen to extreme poverty. This has adversely impacted the nutritional status of the affected population, which in return causes secondary immunodeficiency [12,13]. Tuberculosis transmission is perpetuated by undernutrition, and other conditions associated with socioeconomic and social determinants of health [1,14].

Poverty and undernutrition not only increase vulnerability to the disease, but also delay health seeking among those already sick. It is estimated that undernutrition is responsible for around 20% of the TB incidence in the Region [1,15]. Therefore, the pandemic is also expected to have a significant bearing on the TB epidemic in the SEA Region. In some of the countries of the Region, it is estimated that reduction in body mass index consequent to COVID-19-related undernutrition may cause up to 14% increase in TB incidence among those already vulnerable [15].

While there was a widespread economic downturn during the peak onslaught of COVID-19, the greatest impact on income loss was seen among the poorest, leading to exacerbation of inequity [16]. As per a report published by *Global TB Advocates* that covered SE Asia Region countries, social isolation and lockdowns had increased inequities and human rights-related barriers to access to TB services [17].

In the midst of these challenges, the SEA Region has been at the forefront of galvanizing political commitment. In October 2021, all Member States of the WHO SEA Region committed to renewed response towards ending TB in the COVID-19 era. The Ministerial Statement emphasised on mainstreaming social protection for TB patients as an important intervention to end TB [18]. Countries in the Region have embarked on schemes such as direct benefit transfers and conditional transfers for TB patients.

A modelling analysis has shown that global tuberculosis incidence would be reduced by more than three quarters if poverty was eliminated and, social protection programmes and universal coverage implemented. Implementation of the social protection component alone would reduce tuberculosis incidence significantly [19].

Social protection of TB patients supports alleviation of poverty or susceptibility to poverty by protecting work capacity, avoiding treatment interruption, and preventing loss of income [20]. Social protection for TB patients will also help in emergency preparedness.

To conclude, all TB programmes and partners should work with communities to expand the network of social protection. Free diagnosis and treatment complemented with social protection will help lower catastrophic costs for patients. Mechanisms for social support will not only drive down TB incidence but have a cascading impact on other health programmes.

Financial outlays need to be increased by three times not only to address the current needs of patients but also for future readiness. It is important to address social protection needs of TB patients, not only to achieve the disease-specific UN Sustainable Development Goals but also the overarching goal of eliminating poverty eradication [21]. Countries in the SE Asia Region must bring together partners including communities to design, monitor and implement social protection programmes to ensure availability of adequate resources and sufficient outreach of the programme. All stakeholders should be held accountable to their commitments towards ending TB. The framework for accountability needs to include the contribution towards eliminating catastrophic costs for all patients and ensuring that the biomedical services include elements of counselling, education, and financial and nutrition support for patients.
